# Role of Sensors in Error Propagation with the Dynamic Constrained Observability Method

**DOI:** 10.3390/s21092918

**Published:** 2021-04-21

**Authors:** Tian Peng, Maria Nogal, Joan R. Casas, Jose Turmo

**Affiliations:** 1 Department of Civil and Environmental Engineering, Universitat Politècnica de Catalunya, 08034 Barcelona, Spain; tian.peng@upc.edu (T.P.); joan.ramon.casas@upc.edu (J.R.C.); jose.turmo@upc.edu (J.T.); 2 Faculty of Civil Engineering and Geosciences, Delft University of Technology, 2628 CD Delft, The Netherlands

**Keywords:** system identification, uncertainty quantification, observability, frequencies, mode shapes, epistemic uncertainty, aleatory uncertainty, sensors

## Abstract

The inverse problem of structural system identification is prone to ill-conditioning issues; thus, uniqueness and stability cannot be guaranteed. This issue tends to amplify the error propagation of both the epistemic and aleatory uncertainties, where aleatory uncertainty is related to the accuracy and the quality of sensors. The analysis of uncertainty quantification (UQ) is necessary to assess the effect of uncertainties on the estimated parameters. A literature review is conducted in this paper to check the state of existing approaches for efficient UQ in the parameter identification field. It is identified that the proposed dynamic constrained observability method (COM) can make up for some of the shortcomings of existing methods. After that, the COM is used to analyze a real bridge. The result is compared with the existing method, demonstrating its applicability and correct performance by a reinforced concrete beam. In addition, during the bridge system identification by COM, it is found that the best measurement set in terms of the range will depend on whether the epistemic uncertainty involved or not. It is concluded that, because the epistemic uncertainty will be removed as the knowledge of the structure increases, the optimum sensor placement should be achieved considering not only the accuracy of sensors, but also the unknown structural part.

## 1. Introduction

Numerical or mathematical models are common tools in civil and structural engineering when analyzing the internal forces, the displacements and modal attributes of a structure, or the vibration responses due to dynamic loading. These can be addressed as a direct analysis when the structural parameters are all known. However, given the structural degradation during service life, some structural properties become unknown or uncertain. Structural system identification (SSI), as a one way of inverse analysis, evaluates the actual condition of existing structures, which is of primary importance for their safety.

Most research works focus on the deterministic SSI and probabilistic approach [1,2,3], which aims to find the structural parameters of a numerical model that guarantees the best possible fit between the model output and the observed data. Nevertheless, considering the uncertainties related to the structure model and observed data, uncertainty quantification (UQ) is necessary for assessing the effect of uncertainty and the estimated accuracy [4]. The detail literature review of the UQ approach is given in Section 2.

The observability method (OM) has been used in many fields, such as hydraulics, electrical, and power networks or transportation. This mathematical approach has been applied as a static SSI method [5,6,7,8]. The numerical OM [9] and constrained observability method (COM) [10,11] were developed based on the observability method for the static and dynamic analysis. In order to obtain accurate and reliable parameters, OM identification needs to be robust in terms of variations of systematic modeling uncertainty introduced when modeling complex systems and measurement uncertainty caused by the quality of test equipment and the accuracy of the sensors [12]. Therefore, in order to apply OM accurately and with the required reliability, it is necessary to carry out an UQ analysis. This is the objective of the present paper when dynamic data is used.

UQ analysis seems to be highly probability-independent from optimal sensor placement. In contrast, the sensors need to be installed on the most informative position, that is, the location that provides the least uncertainty in the bridge parameter evaluations [13]. One of the most known and commonly adopted approaches for optimal sensor placement was developed by Kammer [14]. Since then, several variants of this approach have been suggested to resolve the positioning of SSI sensors [13,15,16,17]. However, no research works have noticed that the choice in the best position of the sensors might change when different sources of uncertainty are considered in the uncertainty analysis. To fill this gap, one of the major contributions of this study is to investigate whether there is a best measurement set (optimum sensor deployment providing the most accurate results) independently from the different sources of uncertainty.

This research aims to understand how the uncertainty in the model parameters and data from sensors affect the uncertainty of the output variables, that is, how the uncertainties from different sources propagate or how they will pose their influence on the estimated result. Moreover, by dividing the source of uncertainty into aleatory and epistemic, important insights can be obtained into the extent of uncertainty that can be potentially removed.

Epistemic uncertainty refers to the type of uncertainty caused by the lack of knowledge, thus, with time and more data acquisition, this type of uncertainty can be reduced. On the other hand, the aleatory uncertainty refers to the intrinsic uncertainty that depends on the random nature of the observed property or variable, thus, it cannot be removed no matter the amount of data used [18] as the noise of measurement sensors always exists.

From the practical point of view, determining the level of uncertainty of the estimated parameters through the dynamic observability method is of interest to determine the robustness of the method. Moreover, an informed decision-making process requires not only of a punctual estimation of the variables, but also the level of confidence of the estimation. The uncertainty of the structural parameters will allow a more accurate reliability analysis of the structure. Additionally, it is also essential to compare the advantages and disadvantages of this method with the existing methods to show the applicability of COM.

The motivation of this paper is to check the possibility of gaining insight into the uncertainty quantification before the actual monitoring of a structure. The Dutch bridge known as ‘Hollandse Brug’ is used as an example. This bridge was monitored without a previous evaluation and after its monitoring, the conclusion was that the uncertainty was too big to make any conclusive assessment. What is more, the UQ analysis in the framework of the observability method will be developed to fill the blank of the OM method, and the merit of this COM method for UQ analysis will be discussed.

This paper is organized as follows. In Section 2, an overview of available UQ approaches is given. The principle of the constrained observability method (COM) is described in Section 3. Section 4 presents the case study, the Dutch bridge. The uncertainty analysis considering the effect of aleatory and epistemic by COM is shown in this section, and the analysis conducted to choose the best measurement set of sensors in different scenarios of uncertainty. In Section 5, the comparison of COM and one existing approach, the Bayesian method, is conducted and the discussion of proposed COM. Finally, some conclusions are drawn in Section 6.

## 2. Literature Review of Uncertainty Quantification

The ill-posedness of the inverse SSI problem occurs frequently and SSI is extremely susceptible to uncertainties. Uncertainty quantification is a tool to explore and improve the robustness of the SSI methods. In general, methods for quantifying uncertainty can be divided into two major categories: probabilistic and non-probabilistic approaches. Probabilistic approaches reflect the traditional approach to modeling uncertainty, set on the firm foundations of probability theory, where uncertainty is modeled by appointing unknown quantities to probability density functions (PDFs); these PDFs are then propagated to probabilistic output descriptions. Non-probabilistic methods use random matrix theory to construct an uncertain output of the prediction model operator [4,19].

Non-probabilistic approaches, such as interval methods [20,21,22], fuzzy theory [23] and convex model theory [24], and probabilistic methods, such as the maximum likelihood estimation method [25], Bayesian method [26,27], stochastic inverse method [28], non-parametric minimum power method [29], and probabilistic neural networks [30] have been presented in the existing literature.

In the management of uncertainty, probabilistic Bayesian theory is an attractive framework. It has been widely applied, such as in the identification of material parameters in a cable-stayed bridge [31], plate structures [32], and steel towers [33]. Although the probabilistic method is commonly seen as the most rigorous methodology for dealing with uncertainties effectively and is exceptionally robust to sensor errors [16], it is not especially suitable for epistemic uncertainty modeling [34,35,36]. The argumentation behind this relates to the definition of the (joint) PDFs explaining the unknown quantities: it is argued that adequate qualitative knowledge for constituting a truthful and representative probabilistic model is hardly available. However, model uncertainty has a major effect on estimating structural reliability [37].

To respond to some obvious disadvantages/limitations of the probabilistic approach related to the construction of PDFs and the modeling of epistemic uncertainty, the last few decades have seen an increase in non-probabilistic techniques for uncertainty modeling. It was developed by Soize [19,38,39,40,41], based on the principle of maximum entropy. Most non-probabilistic methods are generated based on the interval analysis. Interval methods are useful to consider the crisp bounds on the non-deterministic values [20]. The non-probabilistic fuzzy approach, an extension of the interval method, was introduced in 1965 by Zadeh [42], aiming to evaluate the response membership function with different confidence degrees [43,44]. Ben-Haim developed the convex model method for evaluating the model usability based on the robustness to uncertainties [45]. Interval approaches, however, are not capable of distinguishing dependency between various model responses by themselves, which may make them severely over-conservative with regard to the real complexity in the responses to the model. Most of the non-probabilistic methods are somehow based on a hypercubic approximation of the result of the interval numerical model, and therefore neglect possible dependence between the output parameters [46,47].

It is worth mentioning that perturbation approaches are proved to be useful for the uncertainty analysis of discrete structural models [48,49,50]. However, this method works well for the aleatory uncertainty (sensitivity to eigenvalues and eigenvectors) but not for epistemic uncertainty.

A probabilistic UQ approach was proposed in this paper to analyze the SSI through the dynamic constrained observability method, by considering both the epistemic uncertainty modeling and the aleatory uncertainty. To overcome some of the drawbacks mentioned above, different modal orders are considered separately, after that, all involved mode orders are put together to estimate the output parameters in an objective function. The method of simultaneous evaluation can appropriately take into account the dependence between various parameters. 

## 3. Dynamic Structural System Identification Methodology

In dynamic SSI by COM, the finite element model (FEM) of the structure has to be defined first. Subsequently, the dynamic equation is obtained with no damping and no external applied forces. For illustration, assume that the system of equations is as follows:

(1) $$K\left\{\emptyset \right\}=\lambda M\left\{\emptyset \right\}$$

In Equation (1), K**,** M**,** λ, and $\left\{\emptyset \right\}$, respectively represent the global stiffness matrix, the mass matrix, squared frequency and mode shape vector. For two-dimensional models with Bernoulli beam elements and $N_{N}$ nodes, the global stiffness matrix K is composed of the characteristic of the beam elements (i.e., length L, elastic moduli E, area A, and inertia I). The mass matrix, M**,** refers either to the consistent mass matrix or to the lumped one. In this paper, the consistent mass matrix was used applying a unit mass density, m. The squared frequency, λ, is considered when the free-vibration happened. ∅ stands for the displacement shapes of the vibrating system, containing the corresponding information, *x*-direction, $u_{ik}$, *y*-direction, $v_{ik}$, and rotation, $w_{ik}$, at each node *k* for each vibration mode *i*. For each node, 3-dof are considered.

In the direct analysis, every element in the matrix K and M  is assumed to be known. The squared frequency, λ, and mode shape vector, ∅, are solved by Equation (1). In dynamic SSI by COM, which is an inverse analysis, the matrix is partially known. Parameters appearing in the matrices K and M are $\left\{E,A,L,I,m\right\}$. It is generally assumed that the length, L, the unit mass density, m, and area, A, are known, whereas elastic moduli, E, and inertia, I, are unknowns. Since the main objective of SSI is to assess the condition of the structure, the estimations of bending stiffnesses, EI, are of primary importance.

Once the unknowns in the matrix K, boundary conditions, $N_{B},$ and measurements are determined, the COM of dynamic SSI can be conducted. Here, the measurement sets are the frequencies and the corresponding partial modal information of the $i^{th}$ vibration mode.

Firstly, the separation of the column of matrix K and M is conducted to place unknown variables of K and M into {∅} for the $i^{th}\;$ vibration mode to form a new matrix $K_{i}^{*}$ and a new matrix $M_{i}^{*}$. After that, the modified modal shapes $\emptyset_{\mathrm{Ki}}^{*}$ and $\emptyset_{\mathrm{Mi}}^{*}$ of the corresponding $K_{i}^{*}$ and $M_{i}^{*}$ include known and unknown terms. Terms of $\emptyset_{Ki,1}^{*sx\times 1}$ and $\emptyset_{Mi,1}^{*nx\times 1}$ are known, whereas $\emptyset_{Ki,0}^{*rx\times 1}$ and $\emptyset_{Ki,0}^{*rx\times 1}$ include unknown terms. $\emptyset_{Ki,0}^{*rx\times 1}$,$\emptyset_{Ki,1}^{*sx\times 1}$,$\;\emptyset_{Mi,0}^{*mx\times 1},\;$ and $\emptyset_{Mi,1}^{*nx\times 1}$ are the partitioned vectors of $\emptyset_{\mathrm{Ki}}^{*}$ and $\emptyset_{\mathrm{Mi}}^{*}$, respectively. The dimensions of each of the elements are given by their superscripts. The modified stiffness and mass matrices, $K_{i}^{*}\;$ and $M_{i}^{*}$, are given the corresponding label according to the split of the modal shapes, as shown in Equation (2). R is the number of measured modes.

Secondly, the system is rearranged in order to have all the unknowns of the system in one column vector, as shown in Equation (3). Thereafter, the product variables are treated as single linear variables to linearize the system for $i^{th}$ vibration mode, such as $\;EI_{j}u_{ik}$*,* $EI_{j}v_{ik},$ and $\;EI_{j}w_{ik}$ instead of “$E\cdot I_{j}\cdot u_{ik}$”, “$E\cdot I_{j}\cdot v_{ik}$”, or “$E\cdot I_{j}\cdot w_{ik}$”.

Thirdly, the equation will be built by combining the information of several models when multiple modal frequencies are considered together. Equation (4) is an example for the first R modal system. Expression in which $B_{i}\;$ is a matrix of constant coefficients,$\;D_{i}\;$ is a fully known vector, and $z_{i}$ contains the full set of unknown variables.

(2) $$K_{i}^{*}\emptyset_{Ki}^{*}=\left[\begin{array}{cc} K_{i,0}^{*\left(3N_{N}-N_{B}\right)\times rx} & K_{i,1}^{*\left(3N_{N}-N_{B}\right)\times sx} \end{array}\right]\left\{\begin{array}{c} \emptyset_{Ki,0}^{*rx\times 1}\\ \emptyset_{Ki,1}^{*sx\times 1} \end{array}\right\}\;=\left[\begin{array}{cc} M_{i,0}^{*\left(3N_{N}-N_{B}\right)\times mx} & M_{i,1}^{*\left(3N_{N}-N_{B}\right)\times nx} \end{array}\right]\left\{\begin{array}{c} \emptyset_{Mi,0}^{*mx\times 1}\\ \emptyset_{Mi,1}^{*nx\times 1} \end{array}\right\}=M_{i}^{*}\emptyset_{Mi}^{*}\;\;\;\left(i=1,2,3\ldots ,R\right)\;\;\;\;$$

(3) $$B_{i}z_{i}=\left[\begin{array}{cc} K_{i,0}^{*\left(3N_{N}-N_{B}\right)\times rx} & -M_{i,0}^{*\left(3N_{N}-N_{B}\right)\times mx} \end{array}\right]\left\{\begin{array}{c} \emptyset_{Ki,0}^{*rx\times 1}\\ \emptyset_{Mi,0}^{*mx\times 1} \end{array}\right\}\;=\left\{\begin{array}{c} M_{i,1}^{*\left(3N_{N}-N_{B}\right)\times nx}\emptyset_{Mi,1}^{*nx\times 1}\;\;\;-K_{i,1}^{*\left(3N_{N}-N_{B}\right)\times sx}\emptyset_{Ki,1}^{*sx\times 1} \end{array}\right\}=D_{i}\;\;\;\;\left(i=1,2,3\ldots ,R\right)$$

(4) $$Bz=\left[\begin{array}{cccc} B_{1} & 0 & 0 & 0\\ 0 & B_{2} & 0 & 0\\ 0 & 0 & \ddots & 0\\ 0 & 0 & 0 & B_{R} \end{array}\right]\left\{\begin{array}{c} z_{1}\\ z_{2}\\ \vdots \\ z_{R} \end{array}\right\}=\left[\begin{array}{cccc} D_{1} & 0 & 0 & 0\\ 0 & D_{2} & 0 & 0\\ 0 & 0 & \ddots & 0\\ 0 & 0 & 0 & D_{R} \end{array}\right]=D\;\;\;\;\;\;\;\;\;\;\;\;\;\;\;\;\;$$

Fourth, the Bz=D is treated as a system of linear equations and its general solution is the sum of a particular solution$,\;z_{p}$, and a homogeneous one$,\;z_{nh}$, which corresponds to the case Bz=0. The general solution is expressed as Equation (5). The value of $\left[V\right]$ is critical for the result of Bz=D. If any row of $\left[V\right]$ is composed by only zeros, then the corresponding particular solution will represent the unique solution of that parameter. The parameter obtained in this step is categorized as an observed parameter. New observed parameters are applied to a next iteration (steps 1–4), until no new parameters are recognized.

(5) $$z=z_{p}+z_{nh}=z_{p}+\left[V\right]\left\{\rho \right\}$$

Lastly, an objective function, Equation (6), is applied to optimize the equation  Bz=D, which is extracted from the last iteration. Here, in order to uncouple the observed variables, the potential implicit condition is constrained in the objective function, i.e.,$\;EI_{j}v_{ik}=EI_{j}*v_{ik}$*,* $EI_{j}w_{ik}=EI_{j}*w_{ik}$.

(6) $$J=W_{\lambda }\overset{R}{\underset{i=1}{\sum }}{\left(\frac{∆\lambda_{i}}{\tilde{\lambda_{i}}}\right)}^{2}+W_{\emptyset }\overset{R}{\underset{i=1}{\sum }}{\left(1-MAC_{i}\right)}^{2}\;$$

(7) $$MAC_{i}\;(\emptyset_{mi},\tilde{\emptyset_{mi}})=\frac{{\left[\emptyset_{mi}^{T}\tilde{\emptyset_{mi}}\right]}^{2}}{\left(\emptyset_{mi}^{T}\emptyset_{mi}\right)\left({\tilde{\emptyset_{mi}}}^{T}\tilde{\emptyset_{mi}}\right)}$$

Equation (6) is used to minimize the squared sum of frequency-related error and mode shape-related error. $∆\lambda_{i}\;$ is the difference between the measured $\tilde{\lambda_{i}}$, and the estimated circular frequencies, $MAC_{i}$ is the modal assurance criterion, which measures the closeness between the calculated mode shape $,\;\emptyset_{mi}$, obtained from the inverse analysis using the estimated stiffnesses and areas, and the measured shape, $\tilde{\emptyset_{mi}}$, as shown in Equation (7). $W_{\lambda }\;\mathrm{and}\;W_{\emptyset }\;$ represent the weighting factors of the mode shape components and circular frequencies coefficient components, respectively. In most analyses, $W_{\lambda }$ and $W_{\emptyset }$ are assumed to be equal [51]. In this paper, the effect of weighting factors was ignored. The specific implementation steps can be found in the literature [11].

## 4. Hollanddse Brug Case Study By COM

### 4.1. Bridge Introduction

**Figure 1:**
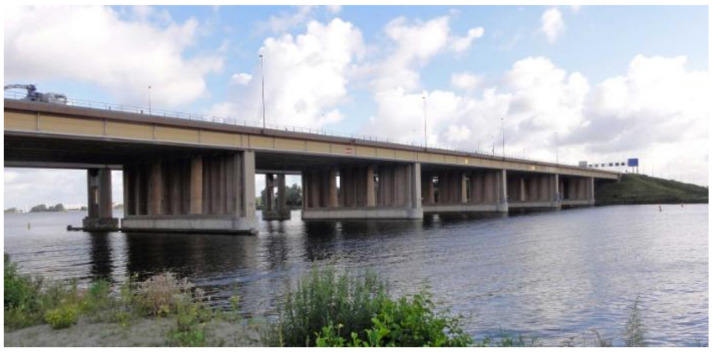
Picture of the Hollandse Brug.

The case study is a prestressed concrete bridge in the Netherlands known as “Hollandse Brug”, see Figure 1. Hollandse Brug, since its opening in 1969, is an important link between Amsterdam and the northeastern area of the country.

The bridge is 355 m long, divided into seven spans of 50.75 m long separated by dilatation joints impeding bending moment transference. Thus, the bridge can be studied by analyzing single-supported beams. Regarding the spans, they are made of nine prefabricated prestressed longitudinal girders of 50.55 m long separated a distance of 4.11 m from each other. Reference [52] includes detailed structural parameters.

**Figure 2:**
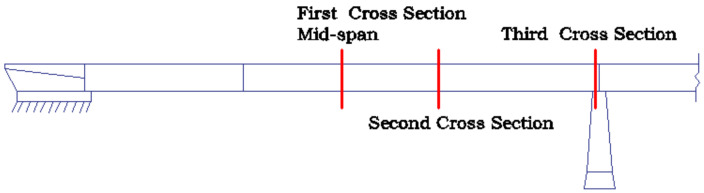
Sensors location of the InfraWatch project.

Structural Health Monitoring (SHM) data were collected for InfraWatch project and obtained from an SHM system installed after the renovation. The SHM system on the Hollandse Brug has strain, vibration, and temperature sensors mounted on three cross-sections of the first span (Figure 2), mid-span (first cross-section), a second cross-section, and a third cross-section (over the bearings). Some of the strain data is available in http://www.infrawatch.com (accessed on 19 April 2021). 

Details about natural frequencies, mode shapes and damping ratios can be collected using vibration sensors placed at different transverse and longitudinal locations of the bridge [52,53,54], and then, the bridge stiffness can be derived through modal data [55,56]. There are many methods to extract modal information, such as the state-space identification, peak-picking method, and frequency domain decomposition [57].

### 4.2. Model Calibration

**Figure 3:**
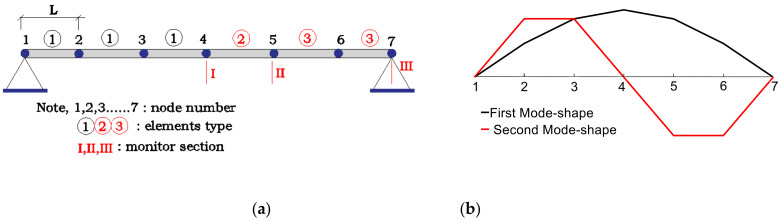
(**a**) First span of Hollandse Brug; (**b**) first and second mode shape.

The unidimensional model of the span of Hollandse Brug is divided into 6 elements (Figure 3). According to the parameters given in References [52,53,54] and model calibration, the simplified model uses the parameters shown in Table 1, obtaining estimations of the frequencies and mode shapes close to the experimental mean data ($f_{1}=2.51\;\mathrm{Hz},\;f_{2}=10.09\;\mathrm{Hz}$). The first and second frequencies match the experimental data correctly with −0.1% and −0.5% errors respectively.

This model will be used as the theoretical representation of the bridge in order to evaluate the effects of uncertainty. The estimated values of the parameters from the analysis done in this paper when sources of uncertainty are considered will be compared with values from Table 1. This will be referred as the theoretical values to be targeted.

### 4.3. Case UQ Analysis

The goal of this section is to assess the uncertainty regarding the estimation of $EI_{2}$ and $EI_{3}$ of the Hollandse Brug when $EI_{1}$ and m are known with some degree of uncertainty. $EI_{1}$, $EI_{2}$ and $EI_{3}$ represent the corresponding values of elements ①, ②, and ③ in Figure 3a.

To assess the uncertainty associated with the output of the structural system identification, the epistemic uncertainty involved in the assumption of the input-parameters (error incurred during the modeling process) and the aleatory uncertainty involved in the measurement error (inaccuracy of sensors) are independently considered. In that way, insights into the contribution of each type of error to the total uncertainty can be obtained. Then, the combined effect is analyzed to determine the total uncertainty of each estimated parameter.

#### 4.3.1. Epistemic Uncertainty: Input-Parameter Errors

The contribution of the errors of the input parameters of the structural model, sometimes referred as model errors, are first analyzed. Here, the effect of boundary conditions was not considered as it is assumed that they were perfectly determined through the model calibration carried out in Section 4.2. In fact, the calibration using the first 2 modal frequencies identified that a pin connection is the correct assumption. In addition, the shear deformation was ignored based on the low value of the ratio cross-section depth to span length. 

Table 2 shows the input parameters considered in this analysis, namely, the mass of the bridge, m, assumed as a constant for the entire bridge, the Young modulus of element type 1, $E_{1}$, and its flexural inertia, $I_{1}\;$ (see Figure 3). The probabilistic distributions assumed to introduce the uncertainty regarding those parameters are also indicated.

They are assumed to follow a normal distribution $\;N\left(u,\delta \right)$, where u is the mean corresponding to the expected value of the variable. The standard deviation, δ, has been chosen to guarantee that the 95% of the distribution falls within the interval $\;\left[u-2\sigma ,\;u+2\sigma \right]$. Thus, the intervals of $m,\;\;E_{1}$ and $I_{1}$ are $\;\left[0.9u\;1.1u\right],\left[0.5u\;1.5u\right]\;\mathrm{and}$ $\left[0.96u\;1.04u\right]$, respectively. The variability in the Young modulus was chosen according to the Reference [58]. All the input parameters are assumed to be statistically independent. It is noted that the uncertainty of the three input parameters of the model can be reduced by conducting non-destructive tests in the bridge.

In order to propagate the uncertainty, Monte Carlo simulation (MCS) was used. MCS requires an input sample made of combinations of realizations of each parameter upon which a model will be evaluated to obtain a sample of the model response. However, this approach may be very time-consuming and for large dimensional problems and some reliability problems, the selected combinations might not yield a response sample that can be considered as a good representation of the population. In other words, relevant information can be dismissed if the input sample is not large enough or not adequately selected. To overcome this issue, several sampling methods have been developed. In this research, the fast optimal Latin hypercube (FOLH) sampling is preferred for its sampling strategy, which can achieve higher sampling accuracy with a smaller sampling scale [59].

**Figure 4:**
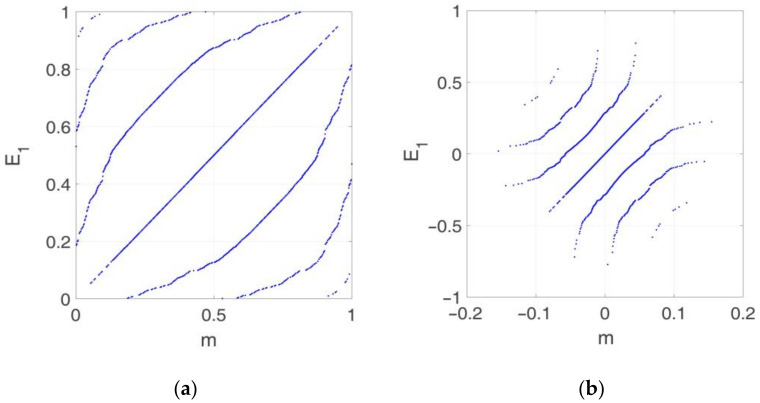
(**a**) Division of the CDFs equally and pairing process; (**b**) resulting sample points.

The FOLH, like the common Latin hypercube (LH) method, requires the selection of the individual realizations of the input parameters according to their probability distribution. To do that, the cumulative distribution function (CDF) of each input parameter was equally divided into the number of required realizations, and then, the corresponding percentile was obtained. By doing so, the set of selected realizations will follow the required probability distribution. The main contribution of FOLH with respect to LH was the way that the realizations were combined (pairing process). To illustrate this process, Figure 4 shows an example considering only two random variables, for instance m and $E_{1}$. Figure 4a depicts the equal division of the CDFs to obtain 10^3^ realizations of each variable. Then the realizations were paired into 10^3^ combinations. Figure 4b shows the resulting sample points. In the case of the variables shown in Table 3, combinations of the three variables should be generated. In this case, a total of 10^3^ sampling points were selected to statistically represent the 3-dimensional space. It is noted that the benefit of the FOLH method was not so obvious in this case, as only three variables were combined. Nevertheless, in the following sections, the number of the involved variables is significantly larger, thus, the FOLH method is required to reduce the computational time without a loss of representation of the input space.

**Figure 5:**
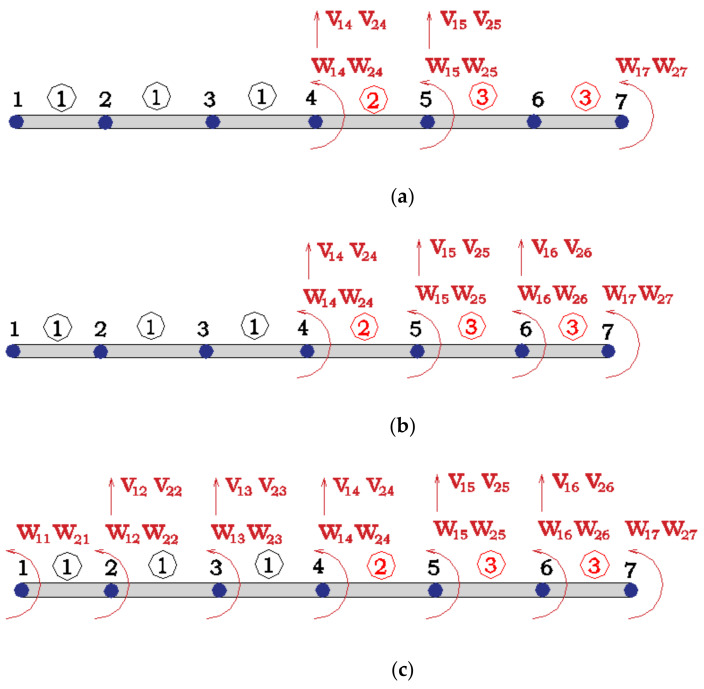
(**a**) Measurement Set A; (**b**) Measurement Set B; (**c**) Measurement Set C.

The sample points were studied for three scenarios that differ in the considered measurement sets. It is noted that in this stage the measurements were assumed error free. The three measurement sets are shown in Figure 5. The symbols $v_{ji}$ and $w_{ji}$ in Figure 5 represent the vertical displacement and rotation of the $j^{th}\;$ node of the $i^{th}\;$ mode shape.

Thus, measurement Set A (Figure 5a) mainly focuses on the estimation of element type 2, the distribution of measurement Set B (Figure 5b) aims at both element types, 2 and 3, and measurement Set C (Figure 5c) includes all the possible measures as it is expected to improve the estimation accuracy of $EI_{2}$ and $EI_{3}$. Given that the corresponding raw row of $\left[V\right]\;$ to $EI_{2}$ and $EI_{3}$ is equal to 0 under these three sets, $EI_{2}$ and $EI_{3}$ can be directly identified by Equation (5), with no need of conducting the optimization step.

**Figure 6:**
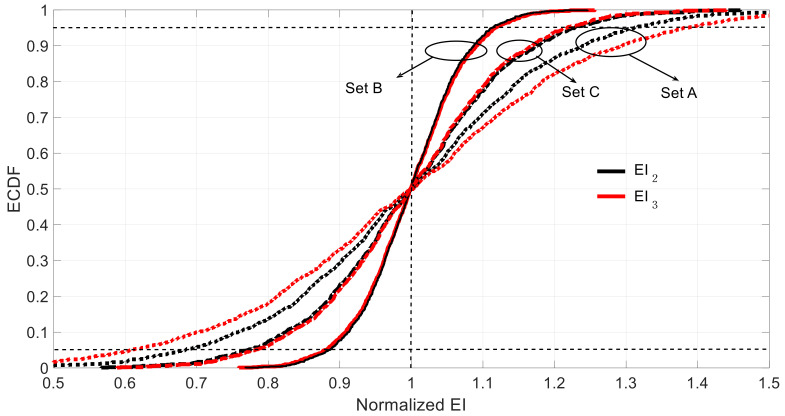
ECDF (empirical cumulative distribution functions) of estimated under different sets considering epistemic uncertainty (the vertical dotted line represents the correct value, and the 5 and 95 percentiles are indicated with horizontal dotted lines).

The results corresponding to the three measurement sets are depicted by their empirical cumulative distribution functions (ECDFs) to avoid making any assumption on the probability distribution of the results. The obtained values, shown in Figure 6, are normalized with respect to the theoretical values. In all the cases, the distributions were almost unbiased and symmetric, which is reflected in the mean and probability of overestimated rows in Table 3.

More precisely, for the measurement Set A, the expected values of the estimated parameters ($EI_{2}$, $EI_{3}$) had 0.0% and 0.3% skewness with respect to the theoretical values, respectively. The 5% and 95% percentages of the normalized values of $EI_{2}$ and $EI_{3}$ were [0.684, 1.312] and [0.608, 1.383], respectively. In absolute terms, $EI_{2}$ will be in the range of [5.57, 10.68]$\times 10^{11}$ and $EI_{3}$ in [4.96, 11.27]$\times 10^{11}$ within 95% confidence interval. It can be seen that the output variable $EI_{2}$ exhibited less uncertainty. This can be explained by the amount of information provided per unit length, which in the case of $EI_{2}$ was bigger than in the case of $EI_{3}$ (see Figure 6).

For the case of the measurement Set B, the skewness and 90% confidence intervals of the normalized $EI_{2}$ and $EI_{3}$ were −0.1% and −0.1% and [0.879, 1.117] and [0.884, 1.113], respectively. In this case, both estimations exhibited the same level of uncertainty. For the measurement Set C, the 90% confidence intervals of the normalized $EI_{2}$ and $EI_{3}$ were [0.770, 1.228] and [0.782, 1.217], which were surprisingly wider than in the case of the Set B even though the Set C contained more information than Set B. This is because of the introduction of redundant information that may derive in some lack of consistency between the mechanical properties of elements ① and the observed displacement and rotation in this part of the structure. In fact, the model to be identified assumed the same mass per unit length all along the span, but not for the stiffness. As no error is assumed in the mode shape measurements (only epistemic uncertainty was considered here) and those were obtained assuming both mass and stiffness uniformly distributed along the span (remember that the modal displacements used in the simulations are obtained with the calibrated model), this produces an inconsistency with the introduction of additional information in Set C. Due to the perfect symmetry and anti-symmetry for modes 1 and 2 respectively under the case of uniformly distributed mass and stiffness along the span, the optimization process does not require information from the half-part of the span. If this information is introduced and does not fit with a symmetrical or anti-symmetrical shape, then the redundant information derives on difficulties in the optimization process and, at the end, on worse (more uncertain) identified parameters.

Therefore, it seems that the best measurement set is B. Table 3 summarizes the discussed results. It is noted that the observed errors can also be affected by the unavoidable computational inaccuracies. As seen in Table 3, the probability of over/underestimation was similar and roughly about 50% in all the cases.

#### 4.3.2. Aleatory Uncertainty: Measurement Errors from Sensors

This part considers the error caused by the accuracy of measurement devices, although the effect of other factors, such as the computational error and the accuracy of the data-extraction method are implicitly included as part of the data processing.

The error assumed for the analysis of this section adopts the values indicated in Table 4. Following the same method as the previous section, $10^{4}\;$ samples are generated for each set with a frequency error level of 3%, a vertical displacement error level of 6%, and a rotation displacement error level of 30%. Normally, the frequency error is small according to the relevant literature [60,61,62], the vertical displacement error range was chosen following Reference [60], who identifies the first vertical displacement with accuracies of about 6%. Given that the accuracy of rotations is lower than the accuracy of vertical displacements [63], 30% was chosen for this purpose.

**Figure 7:**
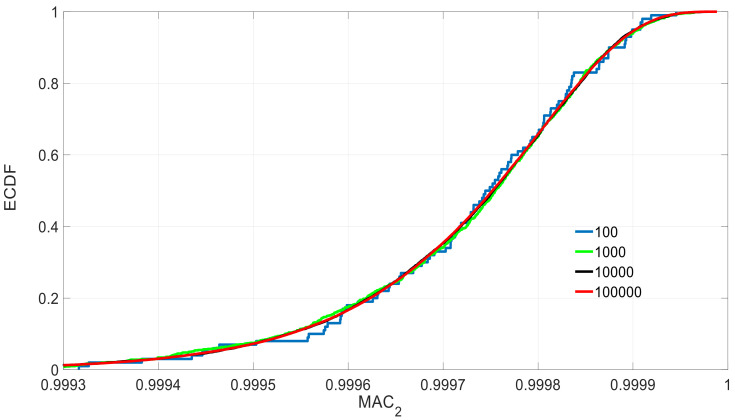
ECDF of $MAC_{2}$ under set C and different sample sizes.

**Figure 8:**
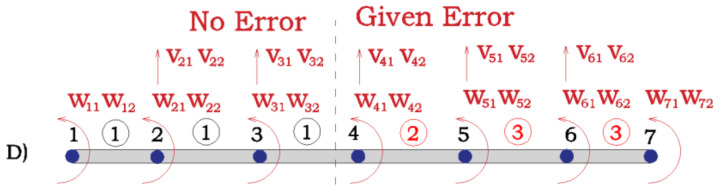
Measurement set D.

The choice of the sampling size is because the number of actual optimization parameters in Equation (6) is 4 when the information of two mode-shapes is used, two frequencies and two MAC. To further check the rationale of this sample size, the $MAC_{1}$ and $MAC_{2}$ are analyzed under different sample sizes and measurement sets. Figure 7 shows an example of the corresponding ECDF under different sample sizes. It shows how the quality of the ECDF for different sample sizes significantly improves till the case of 10^4^. After this, there is not a significant improvement. See how the sample size of 10^4^ was extremely close to the ECDF of 10^5^ in Figure 7. The measurement Set D (Figure 8) is added to compare the effect with three previously defined measurement sets (Figure 5).

**Figure 9:**
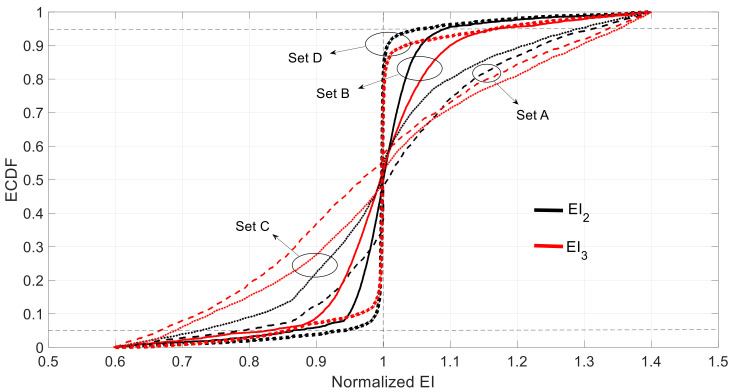
ECDF of estimated under different set considering aleatory uncertainty (the vertical dotted line represents the correct value, and the 5 and 95 percentiles are indicated with horizontal dotted lines).

Figure 9 shows the ECDF of the estimated $EI_{2}$ and $EI_{3}$ under the three measurement sets considering the aleatory uncertainty of the sensor measurements. Table 5 shows 5%, 95% percentages, the bias, standard deviation, and skewness of the estimated data. Here, again, the obtained results show that Set B is the best among the three original sets because it presents the smallest confidence interval, which is non-skewed in the case of $EI_{2}$ and slightly skewed in the case $EI_{3}$ towards conservative values (i.e., underestimate the structural stiffness). Sets A and C exhibit comparable results in terms of confidence intervals. However, the results yielded by Set A are clearly skewed; $EI_{3}$ towards conservative values compensated by $EI_{2}$, which tends to be overestimated under this measurement set. It is recalled that, similarly, Set B presented the most reliable results in terms of epistemic uncertainty, whereas Set A presented the worst estimation. As in the case of epistemic uncertainty, it seems illogical that Set C, which provides more measured data into the system than Set B, provides worse results than Set B. 

In Set C, more measurements corresponding to the left part of the beam are introduced. Error level of the measurements taken from the left and right part of the beam is the same. However, the measurement errors from the left part of the beam have a worse effect on the observed values (corresponding to parameters from the right part of the beam) than the measurement errors from the right part of the beam. In this sense, on the one hand, adding more information should improve the results but on the other hand the errors of this new information are impacting much more the variability and values of the targeted parameters, in such a way that the overall result is worse. This is an interesting and non-intuitive result, as it can be thought that, with the same error level the more measurements, the better and it is not always the case. It is always interesting to add more measurement points, but in the vicinity of the structural part whose mechanical properties are to be identified. This aligns with the fact that where new information without error is introduced (Set D) results from Set B are improved. The most important conclusion of this example is that when the model error is supposed to be low, to decide the sensor locations and, therefore, where to obtain information, it should be taken into account not only the measurement number, but also the structural part whose properties need to be identified. Only in this way, the optimum sensor deployment will be achieved in order to obtain the maximum information (not being redundant) with the minimum uncertainty.

#### 4.3.3. Combination of Epistemic Uncertainty and Aleatory Uncertainty

**Figure 10:**
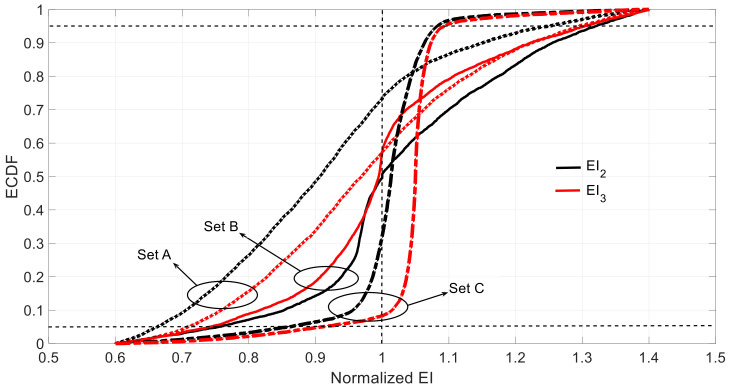
ECDF of estimated under different set considering aleatory and epistemic uncertainties (the vertical dotted line represents the correct value, and the 5 and 95 percentiles are indicated with horizontal dotted lines).

The combination of the two types of errors, i.e., input-parameter error and measurement error, are considered and the three measurement sets shown in Figure 5. The total calculation sample was 10^4^ for each set by the fast optimal Latin hypercube (FOLH) sampling to produce the independent and representative samples and ensure the accuracy of  MAC. The ECDF under this combination is shown in Figure 10, and the related numerical information is illustrated in Table 6.

When both aleatory and epistemic uncertainties were considered, the best measurement set in terms of the uncertainty range was Set C, which includes all the measurement information, instead of Set B that was identified as the best measurement set when considered the uncertainties individually. However, the results from Set C produce some skewness compared with the corresponding value in Table 4 and Table 5, especially for $EI_{3}$, where an overestimation probability of 81.6% is observed. While in terms of structural safety, compared with the huge overestimation of Set C, the results by Set A and Set B tended to be safer with lower percentage of overestimation, the former one performed better on the range and the latter one on the standard deviation. Set B results in the least skewed estimation when compared to the other two sets, while the values of the 5% and 95% percentiles were worse than the ones under Set C. Compared to Figure 6 and Figure 9, the best measurement set in terms of accuracy was Set C rather than Set B, which highlighted the importance of understanding the error source when trying to improve the quality of the estimation. When both model and measurement errors play an important role in the identification process, introducing as many measurements as possible is the best strategy because the information provided by them was not redundant in this case to improve the estimated accuracy. The result for Set C was slightly more biased (compared with the normalized value 1), however, with less uncertainty, as clearly shown by the rows of standard deviation and probability of overestimation in Table 6.

As a summary, it can be concluded that both error sources, epistemic and measurement, interacted in a non-linear manner due to the dynamic effects, in such a way that from the results of their individual effects it cannot be concluded what will happen when both sources act in a combined manner. Hence, to study this, it is necessary to tackle both effects jointly and not in a disaggregate manner.

## 5. Discussion

Hollandse bridge was studied in the InfraWatch project [49,50,51]. After much effort in collecting and analyzing data, no conclusive results were obtained in the structural identification process due to the large level of uncertainty. This fact has motivated the present work, because it is important to know in advance if the uncertainty related to a given SSI approach when applied to a specific structural setup is acceptable or not in real practice. With proper sensor placement, the 90% confidence interval range of the estimated stiffness was found as small as 0.222 for $EI_{2}$ and 0.183 for $EI_{3}$ when considering both sources of uncertainty (Table 6). This means that the estimated stiffness presents around 10% of uncertainty to each direction given that the range was sensibly unbiased. This uncertainty range seems very reasonable if we consider the high level of uncertainty of the input variables (e.g., 50% in the case of the Young modulus or 30% in the rotation of the node of mode shape).

**Figure 11:**
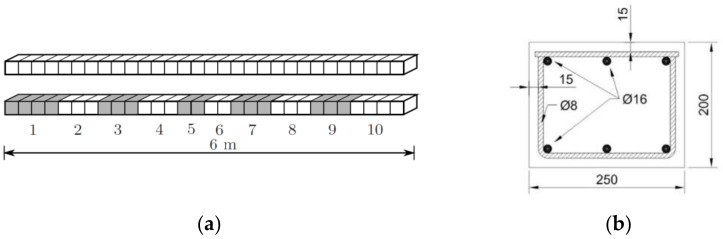
(**a**) Sketch of an reinforced concrete (RC) beam showing 10 sub-elements with 10 different bending stiffness; (**b**) the cross section of the RC beam [4].

**Figure 12:**
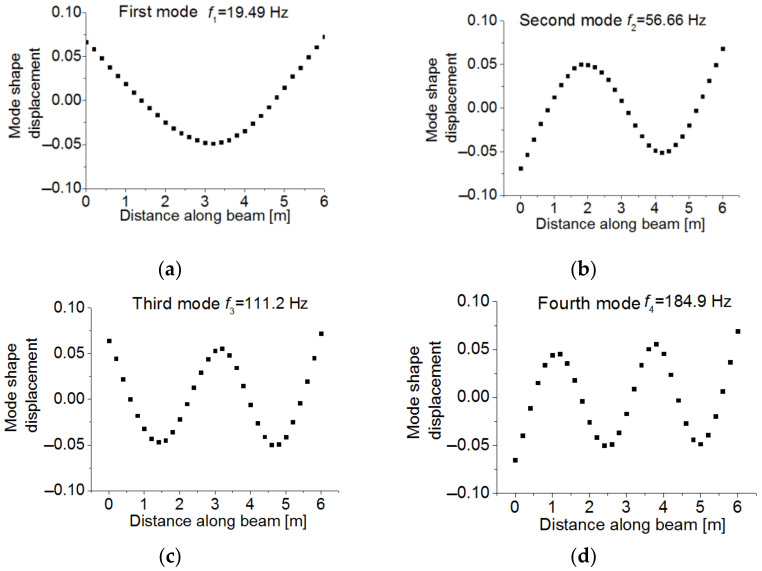
(**a**) The first experimental bending mode and its frequency; (**b**) the second experimental bending mode and its frequency; (**c**) the third experimental bending mode and its frequency; (**d**) the fourth experimental bending mode and its frequency [11].

To assess to which extent the dynamic COM provides acceptable results in terms of uncertainty when compared with other SSI methods in the literature, the example proposed by [4], and further investigated in [11] was used (see Figure 11). This is a reinforced concrete beam with a length of 6 m divided into 10 substructures with a uniform stiffness value, as shown in Figure 11. The measured transverse mode shape displacements were observed at equidistant positions along the beam at 31 points. The resulting mode shape measurements are shown in Figure 12 with their corresponding natural frequencies. The stiffness of these 10 elements given in Reference [4] were taken as the real values for this beam. The considered measurement set includes the frequencies and vertical displacement at the 31 points given by the same reference. Regarding the errors considered, to introduce the epistemic uncertainty, given that it is a free-free vibration beam with unknown stiffness, only the input parameter m is considered. It takes the common density of reinforced concrete $\rho =2551\;\mathrm{kg}/m^{3}$ (probabilistic distribution $N\left(1,\;1*0.05\right)$, the same as in Table 2). The aleatory uncertainty was calculated through the difference between the experimental bending modes and frequencies and the corresponding theoretical data at each of these 31 points. The average values of the obtained uncertainty are given in Table 7.

**Figure 13:**
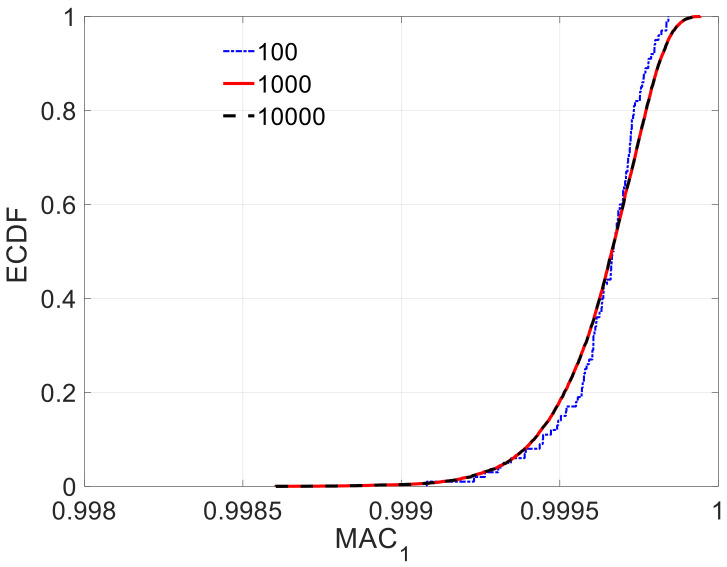
ECDF of $MAC_{1}\;$ under different sample sizes.

**Figure 14:**
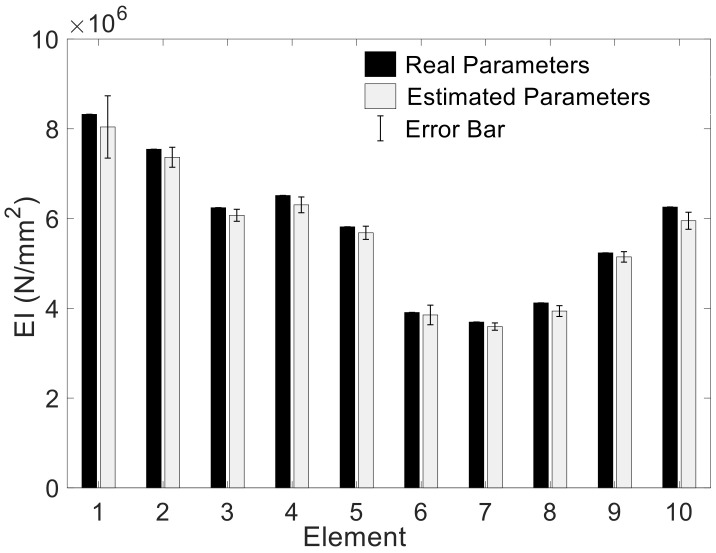
Uncertainty of $EI_{i},\;i=1\sim 10$ given by the mean value and the standard deviation.

**Figure 15:**
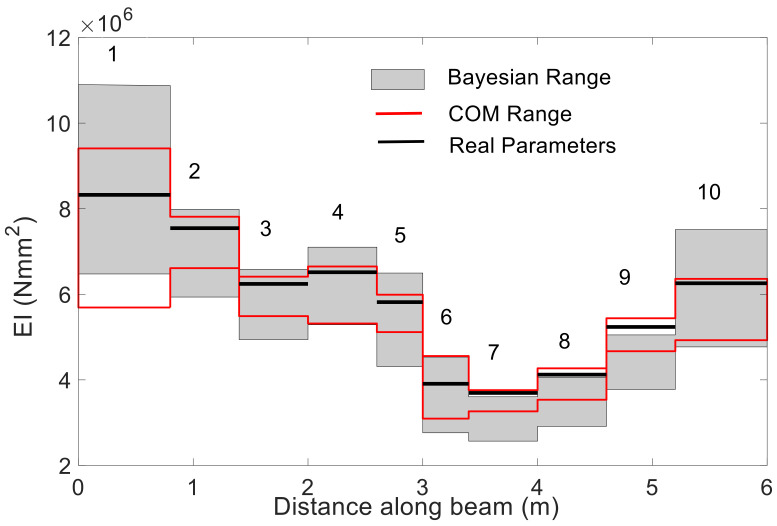
The stiffness range associated with the 99% confidence interval along the beam (the grey shadow represents the result by Bayesian analysis given in [4], the red line represents the range obtained by COM).

**Figure 16:**
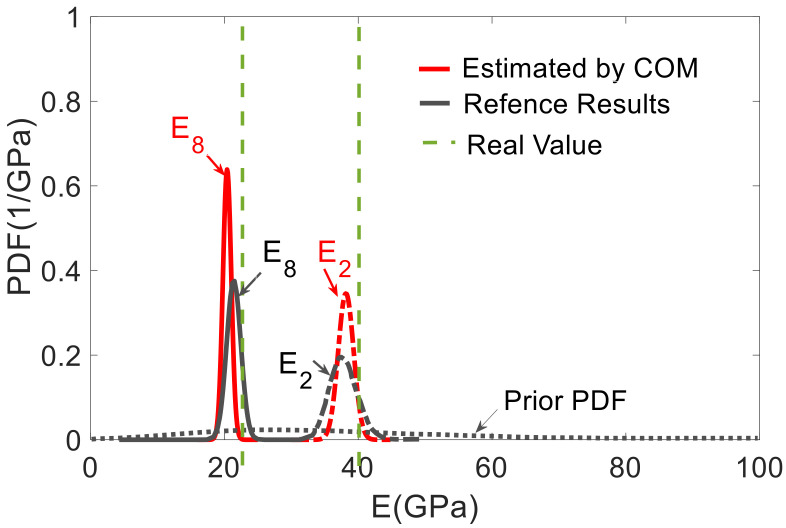
Uncertainty distribution of Young’s modulus $E_{2}$ and $E_{8}$, prior and posterior PDF (grey line) of element Young’s modulus according to [4], the red PDF by COM UQ analysis.

Considering the epistemic and aleatory uncertainty together, the sample size was determined based on the ECDF of $MAC_{i}$, as shown in Figure 13. The $MAC_{i}$ distributions obtained for sample sizes of 10^3^ and 10^4^ were very close to each other, which implies that a sample size of 10^3^ was enough to guarantee the accuracy of $MAC_{i}$. Figure 14 shows the estimated unknown stiffnesses $\;EI_{i},\;\;i=1\sim 10$ and their standard deviation. The COM tended to slightly underestimate the mean values of the stiffness when all mode-shape information was used. The stiffness range associated with the 99% confidence interval obtained by COM was shown in red color in Figure 15, in comparison with the results reported by Simoen when using a Bayesian approach for the SSI (grey shadow). The real values are indicated with a thick black line. For all the elements, COM provides less uncertain estimations. All in all, this figure shows how the UQ associated with COM provides reasonable and acceptable results, and these results were slightly better than the Bayesian approach. Figure 16 depicts the distribution of Young’s moduli $E_{2}$ and $E_{8}$ by the UQ analysis of COM (red line) and the distributions obtained by the Bayesian approach (grey line). It is shown that the proposed approach did not require a prior joint PDF to obtain an accurate stiffness probability distribution.

Even when the obtained uncertainty is acceptable, it is always desirable to minimize such an uncertainty. The analysis of the two sources of uncertainty takes relevance in this context. For instance, it is appreciated that there was no bias and skewness in Table 3 (epistemic uncertainty), whereas obvious bias and skewness is presented in Table 5 and Table 6, which mean these were caused by the sensor error. Thus, increasing the sensor accuracy might reduce the bias and skewness effects. Besides, compared to the estimated data of Sets A, B and C in Table 3, Table 5 and Table 6, the optimal sensor set shifts from Set B under a single source of uncertainty to Set C when considering both uncertainties. This means that selecting the optimal placement of the sensor sets is also an effective method to lower uncertainty of the output in addition to increase the sensor accuracy. However, because the aleatory uncertainty is hard to remove [18], efforts must be made in minimizing the epistemic uncertainty involved in the problem. The more information about the structural setup, the closer the UQ of the SSI will be to the analysis of Section 4.3.2.

## 6. Conclusions

This paper conducted the literature review of UQ analysis to find out the disadvantages of existing methods. The proposed COM method was used to perform the UQ analysis, making up for some drawbacks appropriately. In addition to introducing the basic principles of COM, two sources of uncertainty, that is, epistemic and aleatory, were studied separately and also together to better understand the role of modeling error and measurement error when dynamic COM was used. The following conclusions could be drawn:

1. The analysis of the error propagation in the case of the Hollandse bridge made evident that when the epistemic uncertainty was low (i.e., when very accurate models were used in the identification process), the sensor deployment should take into account not only the measurement accuracy but also the location of unknown structural part. Only in this way, the optimum sensor placement will be achieved in order to obtain the maximum of information (not being redundant) with the minimum uncertainty. Feeding the model with redundant information (if, for instance, the location of sensors is not conveniently chosen) could produce worse results, although more measurement points (more sensors) were deployed.
2. When both epistemic and aleatory uncertainties were relevant, the error propagation decreased with the increase of the measurement points. In this case, the results show that Set B, which includes two additional sensors, was biased to the overestimation side when compared to Set A. If the objective of the identification process was to detect damage, as damage will produce a reduction of the stiffness (due to cracking, for instance), it will be a better solution of the use of fewer sensors, as the trend to the overestimation of the stiffness in the identified elements could hide the existence of damage. This appears as a contradictory conclusion, where the use of an increasing number of sensors derives on decreasing the potentiality of damage detection. However, this result was well in line with the result obtained in the case when only aleatory uncertainty was considered and stated as Conclusion 1, where the addition of more data measurements (Set C compared to Set B) resulted in a worse identification due to the redundancy in the information and the increase in the global measurement error introduced by the additional measurements.
3. The analysis of Hollandse bridge shows that the best measurement set will change from Set B to Set C in terms of range depending whether the epistemic uncertainty was involved or not. Therefore, before the field test execution, when deciding the optimal sensor deployment, it is important to consider the effect of epistemic uncertainty in the sense of trying to gather information from the test that is compatible and non-contradictory with the proposed model. The calculated mode shapes can help on this objective.
4. More accurate estimation of both aleatory and epistemic uncertainty can be obtained with more information about the distribution of the input variables, such as E, m, I (Table 2) and $f_{i},\;v_{ji},\;w_{ji}$ (Table 4).
5. The correct performance of the UQ analysis by COM was verified by an example where the results from the Bayesian method were compared. The performance of the proposed approach was better despite the modeling error in the mass of the structure being considered. The results show the robustness of the method in terms of propagated uncertainty.

## Figures and Tables

**Table 1:** Parameter of each element (deterministic values).

Element Type		Mode Value		
		Length (m/each)	EI (Nm^2^)	Mass (kg/m)
Bridge	1–3	8.425	$8.15\times 10^{11}$	49,000

**Table 2:** Statistical definition of input variables.

List of Variables (Units)	Sampling Size	Probabilistic Distribution	95% Confidence Interval
$m_{1}=m_{2}=m_{3}=m$ $\left(\mathrm{kg}/m\right)$	10^3^	$N\left(49,000,\;49,000\times 0.05\right)$	$49,000\;\left(1\pm 0.1\right)$
$E_{1}$ ($N/m^{2}$)		$N\left(4\times 10^{10},\;4\times 10^{10}\times 0.25\right)$	$4\times 10^{10}\times \left(1\pm 0.5\right)$
$I_{1}\;$($m^{4}$)		$N\left(20.4,\;20.4\times 0.02\right)$	$20.4\;\left(1\pm 0.04\right)$

**Table 3:** Statistical data of the estimated $EI_{2}$ and $EI_{3}$ under different measurement sets (normalized).

	$EI_{2}$			$EI_{3}$		
Measurement Set	A	B	C	A	B	C
p5	0.684	0.879	0.77	0.608	0.884	0.782
p95	1.312	1.117	1.228	1.383	1.113	1.217
p50	1.000	0.999	0.999	1.003	0.999	0.999
Range	0.628	0.238	0.458	0.775	0.229	0.435
Skewness	0.000	−0.001	−0.001	0.003	−0.001	−0.001
Mean (Bias)	0.999	0.999	1.000	0.999	0.999	1.000
Standard Deviation	0.257	0.096	0.185	0.319	0.092	0.176
Probability of Overestimated	49.8%	49.8%	49.8%	49.9%	49.6%	49.8%

**Table 4:** Measurement input variables.

	List of Variables	Sampling Size	Probabilistic Distribution	95% Confidence Interval
Main bridge	Frequencies ($f_{i},\;i=\left\{1,2\right\}$)	$10^{4}$	$N\left(f_{i},\;\;f_{i}\times 0.015\right)$	$\;f_{i}\;\left(1\pm 0.03\right)$
	Vertical displacements ($v_{ji}$)		$N\left(v_{ji},\;v_{ji}\times 0.03\right)$	$v_{ji}\;\left(1\pm 0.06\right)$
	Rotation displacements ($w_{ji}$)		$N\left(w_{ji},\;\;w_{ji}\times 0.15\right)$	$w_{ji}\;\left(1\pm 0.3\right)$

**Table 5:** Statistical data of the estimated $EI_{2}$ and $EI_{3}$ under different measurement sets.

	$EI_{2}$				$EI_{3}$			
Measurement Set	A	B	C	D	A	B	C	D
p5	0.788	0.865	0.728	0.936	0.666	0.827	0.670	0.857
p95	1.313	1.092	1.292	1.060	1.336	1.169	1.352	1.165
p50	1.006	1.000	0.997	0.998	0.968	0.995	1.000	0.999
Range	0.525	0.227	0.564	0.124	0.67	0.342	0.682	0.308
Skewness	0.006	0.000	0.003	0.002	−0.032	−0.005	0.000	0.001
Mean (Bias)	1.032	1.003	0.980	0.998	0.997	1.000	1.007	0.997
Standard Deviation	0.144	0.064	0.195	0.053	0.152	0.099	0.191	0.087
Probability of Overestimated	56.8%	50.1%	49.8%	50.2%	45.7%	49.8%	49.5%	51.2%

**Table 6:** Statistical data of the estimated $EI_{2}$ and $EI_{3}$ under different measurement sets.

	$EI_{2}$			$EI_{3}$		
Measurement Set	A	B	C	A	B	C
p5	0.663	0.754	0.860	0.709	0.742	0.911
p95	1.250	1.320	1.080	1.295	1.304	1.094
p50	0.906	1.000	1.013	0.9691	0.9946	1.050
Range	0.587	0.566	0.222	0.586	0.562	0.183
Skewness	−0.094	0.000	0.013	−0.031	−0.005	0.050
Mean (Bias)	0.915	1.032	1.006	0.979	1.003	1.042
Standard Deviation	0.168	0.158	0.079	0.172	0.152	0.072
Probability of Overestimated	26.6%	49.9%	67.8%	42.8%	42.8%	81.6%

**Table 7:** Measurement input variables (averaged values for the 31 measured points).

	List of Variables	ProbabilisticDistribution	95% Confidence Interval
Structure in Figure 11	Frequencies ($f_{i},\;i=\left\{1,4\right\}$)	$N\left(f_{i},\;\;f_{i}\times 0.005\right)$	$\;f_{i}\;\left(1\pm 0.01\right)$
	Vertical displacements ($v_{ji}$)	$N\left(v_{ji},\;v_{ji}\times 0.03\right)$	$v_{ji}\;\left(1\pm 0.06\right)$

## Data Availability

Not applicable.
